# Gender-Dependent Differences in Plasma Matrix Metalloproteinase-8 Elevated in Pulmonary Tuberculosis

**DOI:** 10.1371/journal.pone.0117605

**Published:** 2015-01-30

**Authors:** Tarangini Sathyamoorthy, Gurjinder Sandhu, Liku B. Tezera, Richard Thomas, Akul Singhania, Christopher H. Woelk, Borislav D. Dimitrov, Dan Agranoff, Carlton A. W. Evans, Jon S. Friedland, Paul T. Elkington

**Affiliations:** 1 Infectious Diseases and Immunity, Imperial College London, London, United Kingdom; 2 NIHR Southampton Respiratory Biomedical Research Unit, Clinical and Experimental Sciences Academic Unit, Faculty of Medicine, University of Southampton, Southampton, United Kingdom; 3 The Royal London Hospital, Whitechapel, United Kingdom; 4 Primary Care and Population Sciences, Faculty of Medicine, University of Southampton, Southampton, United Kingdom; 5 Brighton and Sussex University Hospitals NHS Trust, Brighton, United Kingdom; 6 Microbiology, Universidad Cayetano Heredia, Lima, Peru; 7 Institute for Life Sciences, University of Southampton, Southampton, United Kingdom; INRS, CANADA

## Abstract

Tuberculosis (TB) remains a global health pandemic and greater understanding of underlying pathogenesis is required to develop novel therapeutic and diagnostic approaches. Matrix metalloproteinases (MMPs) are emerging as key effectors of tissue destruction in TB but have not been comprehensively studied in plasma, nor have gender differences been investigated. We measured the plasma concentrations of MMPs in a carefully characterised, prospectively recruited clinical cohort of 380 individuals. The collagenases, MMP-1 and MMP-8, were elevated in plasma of patients with pulmonary TB relative to healthy controls, and MMP-7 (matrilysin) and MMP-9 (gelatinase B) were also increased. MMP-8 was TB-specific (p<0.001), not being elevated in symptomatic controls (symptoms suspicious of TB but active disease excluded). Plasma MMP-8 concentrations inversely correlated with body mass index. Plasma MMP-8 concentration was 1.51-fold higher in males than females with TB (p<0.05) and this difference was not due to greater disease severity in men. Gender-specific analysis of MMPs demonstrated consistent increase in MMP-1 and -8 in TB, but MMP-8 was a better discriminator for TB in men. Plasma collagenases are elevated in pulmonary TB and differ between men and women. Gender must be considered in investigation of TB immunopathology and development of novel diagnostic markers.

## Introduction

Tuberculosis (TB) remains a severe global health problem with 9.0 million new cases and 1.5 million deaths in 2013 [1]. Multidrug resistant and extensively drug-resistant TB cases are rising [1] and “totally drug-resistant TB” has been reported in Iran, India and South Africa [2]. To develop new therapeutic and diagnostic approaches, it is critical to understand underlying mechanisms of disease. Pulmonary tissue damage is characteristic of tuberculosis and is a consequence of immunopathology driven by the host response to *Mycobacterium tuberculosis (Mtb)*. Pulmonary extracellular matrix (ECM) breakdown and subsequent cavitation results in morbidity, mortality [3] and facilitates transmission [4–6]. TB is more common in males than females, with a 1.9-fold excess in case notification globally, which may result from both sociological and biological factors [7–10]. However, although some evidence of biological differences in TB between men and women exists, this has historically been overlooked by the field [7,11].

The matrix metalloproteinases (MMPs) are proteases able to degrade all components of the pulmonary extracellular matrix [12,13] and therefore are implicated in the pathology of cavitary pulmonary TB [14]. Accumulating evidence implicates the MMPs in tissue destruction in TB, in particular MMP-1 driving collagenolysis [15,16] and MMP-9 regulating cellular recruitment to the granuloma [17]. MMP-8 and MMP-9 are principally derived from neutrophils and are contained within granules [18]. MMP-8 (neutrophil collagenase) can degrade collagen and has been implicated in other destructive lung pathologies [19]. Neutrophils are relatively unique in that they store pre-synthesised MMP-8, and neutrophils are emerging as key pathological mediators at the time of TB diagnosis [20,21]. Circulating MMPs have not previously been systematically investigated in TB, in particular including the important control group of respiratory symptomatics (patients with symptoms suspicious of TB but sputum culture negative). MMPs are most appropriately analysed in plasma samples [22] and concentrations have been reported to differ between men and women [23].

Analysis of circulating inflammatory mediators in TB, such as cytokines and chemokines, in plasma and serum to define underlying mechanisms of pathogenesis has generally shown surprisingly small differences between patients with TB and control groups [24,25]. In addition, these investigations generally do not consider gender differences [24]. We hypothesised that MMPs, as final effectors of immunopathology in TB, may identify greater divergence between groups. We analysed plasma concentrations of MMPs in a prospectively collected cohort of 380 patients with active tuberculosis, healthy controls, and the key comparator group of respiratory symptomatics. We demonstrate that circulating collagenases are elevated in TB, further implicating collagenase activity in driving immune-mediated tissue damage, and identify MMP-8 as a novel marker of TB compared to other respiratory infections. MMP-8 concentrations are higher in men than women with TB, highlighting a previously overlooked potential confounder in the investigation of TB pathology and assessment of novel diagnostic strategies.

## Methods

### Ethics statement

All participants gave written informed consent and the research was approved by internationally accredited ethics committees at Universidad Peruana Cayetano Heredia (Lima, Peru) and Imperial College London (London, United Kingdom).

### Study design

SELDI-TOF proteomic analyses of a subset of these plasma samples along with baseline clinical characteristics of those patients have previously been reported [26]. Participants were recruited over a 2 year period from 16 community TB clinics serving the shantytown of Ventanilla in Lima, Peru. The clinical management of patients was not altered from the standardised regime, and all patients provided four consecutive sputum samples for microscopy and culture. Patients with active TB were recruited on the basis of positive sputum microscopy with subsequent confirmation by culture. Symptomatic controls were those who had symptoms suspicious of TB (persistent cough and 2 or more features from fever, weight loss, decreased appetite or haemoptysis) but in whom active TB was subsequently excluded by sputum microscopy and negative sputum culture. This group was deemed to have an alternative respiratory infection and described as “respiratory symptomatics”. Healthy controls were recruited from randomly selected households in the community where patients with active TB were not resident. Informed consent was given by or on behalf of all participants.

### Sample collection

A 4 ml blood sample was obtained from each participant either before initiation of treatment or within 48 hours of starting treatment. Blood was collected in EDTA and transferred to the laboratory on ice. The plasma was separated (3500 rpm, 10 minutes), aliquotted in 1.5ml cryovials and frozen at -70°C for subsequent analysis [26].

### Luminex assay

The samples were defrosted at 4°C and 50μl aliquots were placed in 96 well plates before refreezing at -70°C. This minimized freeze thaw cycles of the samples prior to analysis. Analysis used MMP beads (R&D systems, Abingdon, UK) on the Luminex 200 platform (Biorad Bioplex 200, Hemel Hempstead), as per manufacturer’s instructions for MMP-1, -2, -3, -7, -8, -9, -10, -12 and-13. The lower level of detection of the assay was 1.1, 12.6, 7.3, 6.6, 16.6, 13.7, 3.2, 0.7 and 63.5pcg/ml respectively. In preliminary studies, MMP-12 and MMP-13 were not detected in plasma of either control or TB patients and so were not studied further. MMP-2 was detected but was excluded from statistical analysis as it was present in the plasma at high baseline concentrations and mean fluorescence intensity (MFI) values were predominantly above the assay range.

### Statistical analysis

The luminex output was exported into Microsoft Excel and a master data file compiled integrating the clinical characteristics with laboratory values. Initial statistical analysis was performed using GraphPad Prism 5. Mann Whitney U test was used to compare paired values and One Way Analysis of Variance (ANOVA) with Tukey’s post hoc analysis for multiple comparisons. The multivariate analysis using a stepwise logistic regression conditional model was performed in SPSS version 22.

Principal component analysis (PCA) was conducted using the FactoMineR / scatterplot3d / rgl packages in R version 3.0.2. To account for missing values within the dataset, the missMDA package in R was used, which performs imputation for missing values with multivariate data analysis methods. ROC curves were generated using R version 2.15.2 using the package pROC and Youden’s index was used to calculate an optimal cut off threshold in each case.

## Results

### Baseline patient characteristics

120 healthy controls, 109 symptomatic controls and 151 patients with active pulmonary TB were recruited (clinical characteristics of cohort, Table 1). The groups were well matched for age, but there were higher proportions of females in the healthy and symptomatic controls group (66% for both) and a higher proportion of males in the active TB group (56%). All the patients with active TB were either smear or culture positive with 92% smear and culture positive. All symptomatic controls were smear and culture negative. There was a lower proportion of patients with past TB in the healthy controls (3%) but similar proportion in the symptomatic controls (17%) and active TB (23%). Patients with active TB had lower body mass index (BMI) at enrolment compared with symptomatic controls and healthy controls (21.66 *vs*. 24.15 and 25.86 respectively). Symptomatic controls and patients with active TB had overlapping clinical symptoms, although the duration was longer in the TB group (median 7 days *vs*. 2 days).

**Table 1:** Demographic and clinical characteristics.

Patient characteristics	Healthy controls		Respiratory Symptomatics		TB	
	n	%	n	%	n	%
Patients	120	31	109	29	151	40
Age (years)*	31 (23–40)		32 (24.5–47.5)		28.5 (22–38)	
Sex ratio Male: Female	41:79	34:66	37:72	34:66	85:66	56:44
Sputum smear						
Positive	-	-	0	0	139	92
Negative	-	-	109	100	12	8
Sputum culture						
Positive	-	-	0	0	139	92
Negative	-	-	109	100	12	8
History of past TB	4	3	19	17	34	23
BMI**	25.86 (4.33)		24.15 (4.67)		21.66 (2.97)	
Presence of symptoms / signs						
Cough	42	35	109	100	154	100
Haemoptysis	2	2	28	26	71	47
Fever	17	14	52	48	93	62
Weight loss	40	33	72	66	123	81
Appetite loss	23	19	57	52	109	72
Symptoms duration (days)*	0		2 (1–10)		7 (1–30)	

Abbreviations: IQR—Interquartile range, BMI—Body Mass Index.

*Median (IQR);

**Mean (SD)

### Plasma MMP-1 and MMP-8 are elevated in pulmonary TB and MMP-8 is TB-specific

**Fig 1:**
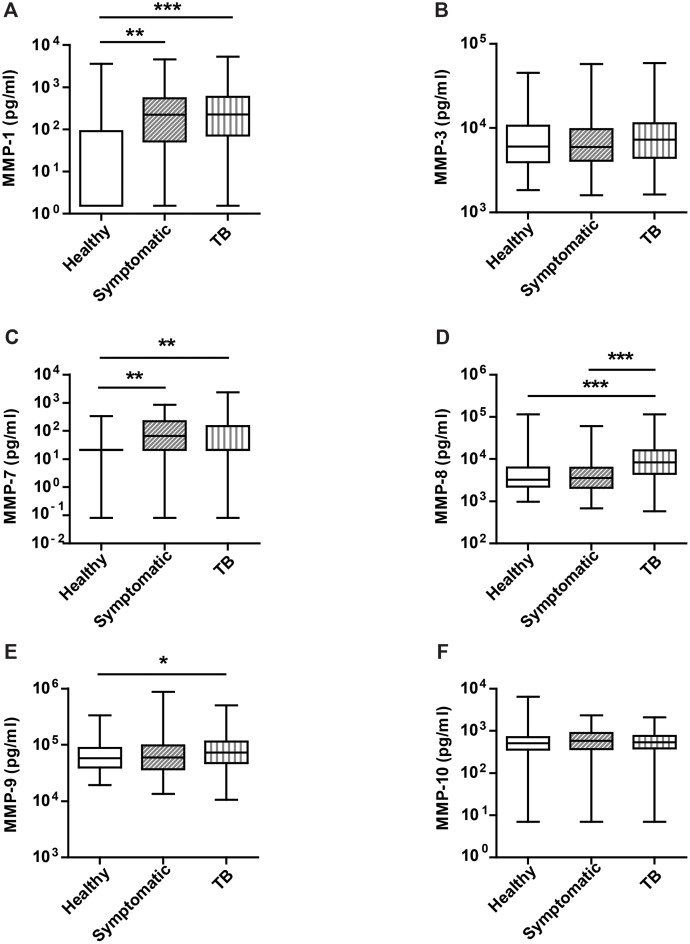
Neutrophil collagenase MMP-8 is specifically upregulated in the plasma in active pulmonary tuberculosis (TB). Plasma samples were collected prospectively from patients in Lima, Peru. MMP concentrations were measured by Luminex bead array. **A**. MMP-1 was increased in both active TB and respiratory symptomatics compared to healthy controls (p<0.001 and 0.01 respectively). **B**. MMP-3 showed no difference between the groups. **C**. MMP-7 was increased in both active TB and symptomatic controls compared to healthy controls (p<0.01 for both). **D**. MMP-8 was specifically increased in active TB compared to both respiratory symptomatics and healthy controls (p<0.001 for both). **E**. MMP-9 was increased in active TB only compared to healthy controls (p<0.05). **F**. MMP-10 showed no difference between the groups. Statistical analysis was performed using a one way ANOVA with Tukey’s post hoc test, ***p<0.001, **p<0.01, *p<0.05. Each box represents the 25^th^ to 75^th^ centiles, the central line the median, and the whiskers the minimum and maximum values.

We analysed concentrations of MMP-1, -3, -7, -8, -9 and, -10 in the plasma of all patients. MMP-1 (interstitial collagenase) was increased in both active pulmonary TB and respiratory symptomatics compared with healthy controls (*p*<0.001 and <0.01 respectively), but there was no difference between active TB and respiratory symptomatics (Fig. 1A). MMP-8 (neutrophil collagenase) was specifically increased in the plasma of patients with active TB compared to both respiratory symptomatics and healthy controls (Fig. 1D, both p <0.001). There was no significant difference between symptomatics and healthy controls. MMP-7 (matrilysin) was increased in both active pulmonary TB and symptomatics compared healthy controls (p <0.01 for both), but there was no difference between active TB and symptomatics (Fig. 1C). However, the median MMP-7 value in respiratory symptomatics was higher at 66.3pg/ ml compared to 20.1pg/ml in both pulmonary TB and healthy controls, suggesting that plasma MMP-7 is elevated in a small proportion of patients with TB. MMP-9 was increased in active TB only compared to healthy controls (Fig. 1E, p<0.05). There was no statistically significant difference in MMP-9 between active TB and symptomatic controls or symptomatic controls and healthy controls. MMP-3 and MMP-10 showed no difference between any of the groups (Fig. 1B and 1F).

**Fig 2:**
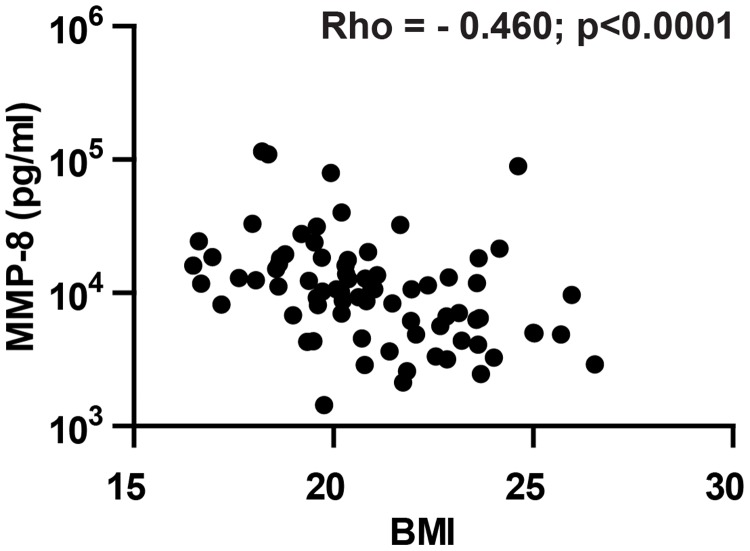
Plasma MMP-8 concentration inversely correlates with BMI. Analysis of plasma MMP-8 concentration and BMI shows an inverse relationship by Spearman’s correlation (Rho = -0.460, p <0.001).

Within the control group, patients were classified as either having latent TB, with a positive interferon gamma release assay (IGRA), or not TB-exposed, with a negative IGRA. Analysis of control patients according to IGRA status did not demonstrate significant differences for the majority of MMPs (Fig. A in S1 File), but plasma MMP-7 concentrations were significantly elevated in patients with latent TB. When MMP concentrations were analysed against baseline clinical characteristics, the strongest association was an inverse correlation between plasma MMP-8 and body mass index (Fig. 2, rho-0.460).

### Plasma MMP-8 is higher in males with TB than females with TB

**Fig 3:**
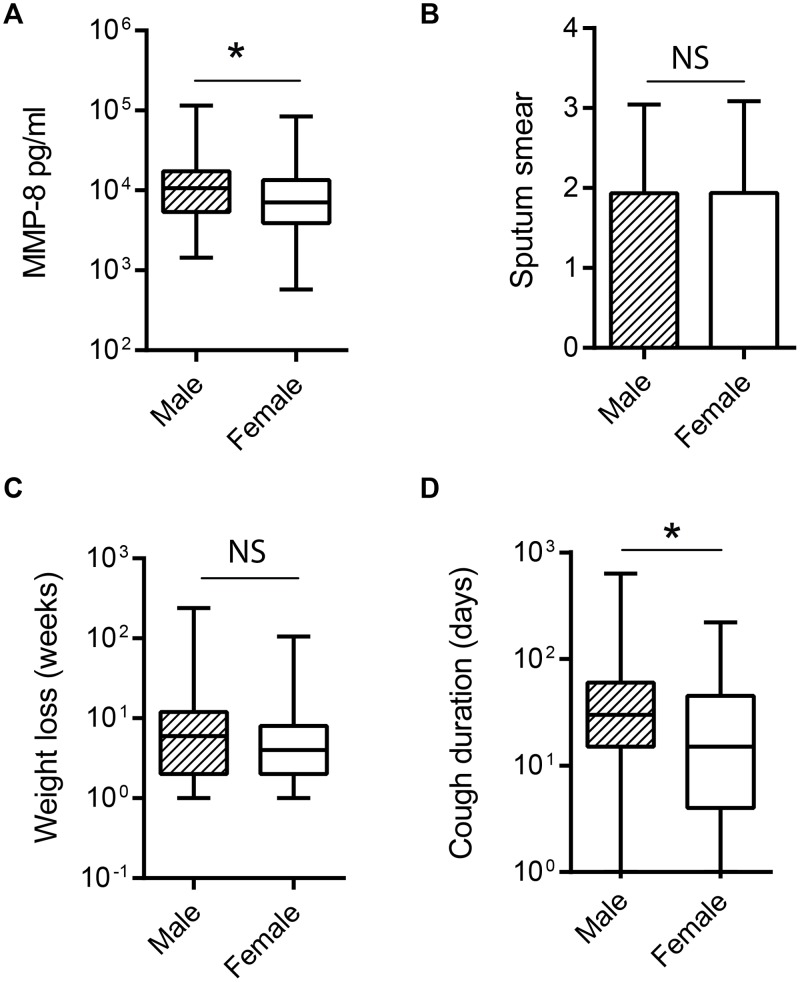
Analysis of clinical and laboratory parameters in patients with TB by gender. **A**. Amongst patients with TB, MMP-8 was analyzed by gender. Plasma MMP-8 concentrations were higher in males than females. **B**. There was no significant difference in sputum mycobacterial load between genders as analyzed by sputum smear quantitation. **C**. There was no difference in duration of weight loss between men and women with TB. **D**. Men with TB had a greater duration of cough than women at time of diagnosis of TB. Statistical analysis was performed using Mann-Whitney U Test, *p<0.05, NS Not significant.

Previously, MMP-3, MMP-8 and MMP-9 have been observed to be higher in males than in females [23]. Therefore, we analysed MMPs according to gender. In our cohort, MMP-1, MMP-3, MMP-8 and MMP-9 were higher in males when all patients were analysed together. In the cohort, the active TB group had a higher proportion of males while symptomatic controls had more females, potentially introducing a confounder, and therefore MMP concentrations were analysed in patients with TB only. In the TB group, MMP-8 was significantly elevated in males versus females (Fig. 3A, p = 0.021). The increased MMP-8 concentrations may result either from males presenting with more severe disease, or alternatively, MMP-8 concentrations being independently higher in males with TB. To address this question both stratified analysis and multivariate analysis were performed. In the stratified analysis, we compared the sputum AFB load, duration of weight loss and duration of cough between genders only in the patients with TB, to determine if males had more severe or prolonged disease. There was no difference in sputum smear or weight loss between genders (Fig. 3B and Fig. 3C), but cough duration in males was more prolonged (Fig. 3D), which may reflect either greater pulmonary pathology in men or delayed presentation.

Analysis of the relationship between plasma MMP-8 concentrations and sputum smear, duration of weight loss or duration of cough did not demonstrate any significant correlations, again indicating that elevated MMP-8 concentrations were not due to greater disease severity or delayed diagnosis. In a multivariate analysis, we analysed patients with TB versus controls and patients with respiratory symptomatics and performed a backward stepwise logistic regression model to determine whether these variables are independent predictors of TB (Table 2). We found that MMP-8 is an independent predictor of TB status as adjusted for gender, duration of weight loss and duration of cough as markers of the duration/severity of the disease. Gender was an independent predictor in this model, again demonstrating that the elevated MMP-8 concentrations in males were not due to delayed presentation.

**Table 2:** Multivariate analysis of TB status.

Variable	Odds ratio Exp (B)	95% CI for EXP (B)		p-value
MMP-8* (pg/ml)	2.73	2.05	3.64	<0.001
Gender (M/F)	1.84	1.13	2.98	0.014
Duration of cough (days)	1.01	1.00	1.02	0.011
Weight loss (weeks)	1.02	0.99	1.04	0.067**

A backward stepwise logistic regression model was built to predict TB status in 380 patients using plasma MMP-8 concentration as adjusted for gender, duration of cough and duration of weight loss as covariates.

*Log-transformed values of MMP-8 are used.

**Marginal significance.

**Fig 4:**
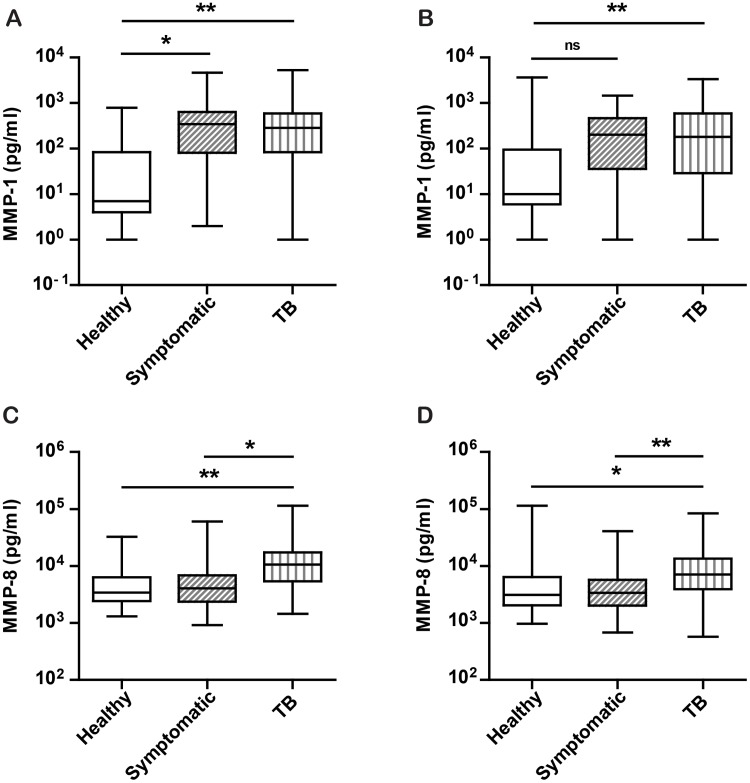
Plasma MMP-1 and MMP-8 remain elevated in TB when analysed by gender. As there was a higher proportion of males in the TB group, plasma MMP-1 and MMP-8 concentrations were analysed individually for males and females in each group. **A**. In males, MMP-1 was increased in active TB and symptomatics compared to healthy controls. **B**. In females, MMP-1 was only increased in active TB compared to healthy controls. **C, D**. In males (C) and females (D), MMP-8 was specifically increased in active TB only compared to symptomatic controls and healthy controls. Statistical analysis was performed using a one way ANOVA with Tukey’s post hoc test, **p<0.01, * p<0.05.

Next, we analysed plasma MMPs individually for each gender. MMP-1 remained increased in both active TB and symptomatics compared to healthy controls for males (Fig. 4A, p<0.01 and <0.05 respectively), but in females MMP-1 was only increased in active TB compared to healthy controls (Fig. 4B, p<0.01). MMP-8 remained increased in active TB compared to both symptomatics and healthy controls in males (Fig. 4C, p< 0.05 and <0.01 respectively) and females (Fig. 4D, p < 0.01 and <0.05 respectively). MMP-3 and MMP-9 showed no significant differences between the three clinical groups for the sexes when analysed separately. The median values and interquartile ranges for MMP-1, MMP-3, MMP-8 and MMP-9 for the sexes separately and across the three clinical groups are provided in Table A in S1 File.

### Principal component analysis demonstrates distinction between TB and control patients but not respiratory symptomatics

**Fig 5:**
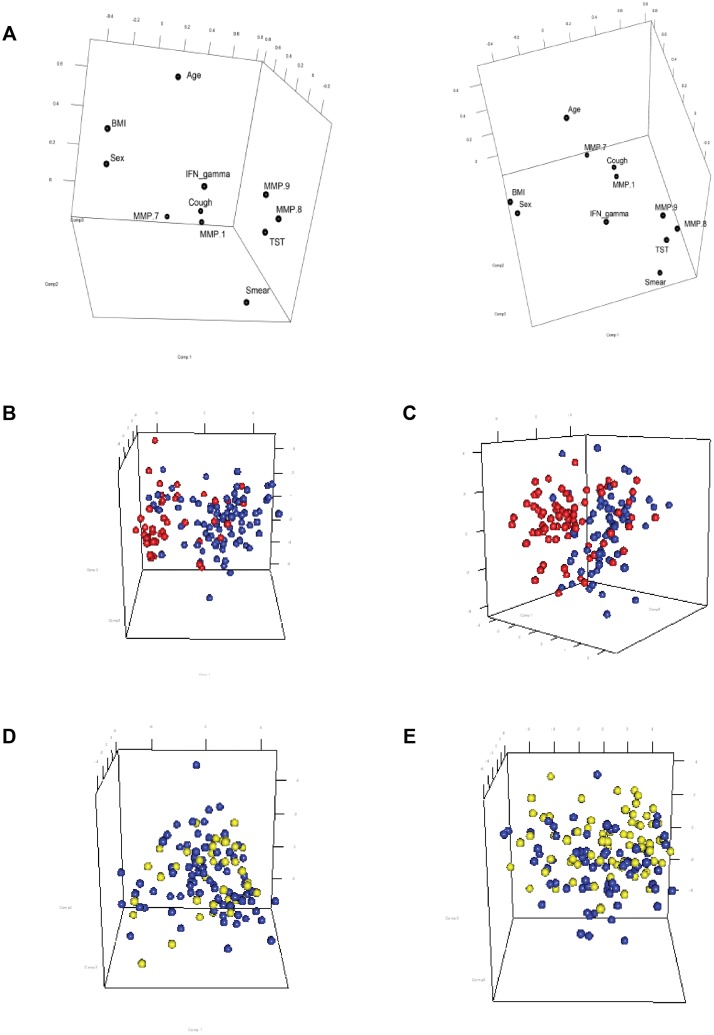
Prinicipal component analysis (PCA) demonstrates discrimination between TB and controls, but not between TB and respiratory symptomatics. **A**. Three dimensional PCA plot demonstrating the association between different analytes. Individual points represent a single analyte and the distance between points reflects their Pearson correlation coefficient, whereby a short distance demonstrates close correlation. Two projections are shown purely for visualization purposes, demonstrating close correlation between MMP-8 and 9, BMI and sex, and MMP-1 and cough respectively. **B-E**. Three dimensional network principal component analysis plots generated using key parameters that differed between patients with TB and controls. Measurements for MMP-1, MMP-7, MMP-8, MMP-9, BMI and IFN-γ were log_2_ transformed and visualized for each gender using PCA plots using the rgl package in R. The colour of the circle represents patient status, red indicating healthy controls, blue TB and yellow respiratory symptomatics. Distinction between patient groups is seen between healthy controls and TB for males (B) and females (C), but between TB and respiratory symptomatics there is much greater overlap (D, males, E, females).

We next investigated association between key clinical features of TB documented at time of recruitment and plasma MMP concentrations by principal component analysis (PCA). First, we analysed the relationship between variables when principle components are calculated based on their values across samples, analysing circulating MMPs and clinical characteristics such as cough, body mass index (BMI), sex, sputum smear result, tuberculin skin test (TST) and interferon-gamma release assay (IGRA). MMP-1, -7, -8 and-9 were included in the analysis as they significantly differed in the initial plasma analysis (Fig. 5A). MMP-8 and-9 cluster together, suggesting that both have a common source and these proteases are co-secreted by neutrophils. BMI and sex were similarly closely associated, as would be predicted from anthropometry. MMP-1 associated with cough, which may imply a mechanistic link.

Next, we performed analysis of the relationship between samples when principle components are calculated based on the variable values in each sample. We combined discriminatory clinical and laboratory continuous variables (BMI, IGRA concentration, MMP-1, MMP-7, MMP-8 and MMP-9) to determine whether this analytical approach differentiated between patient groups. As MMPs differed between gender, analysis was performed separately for males and females. Comparison between healthy controls and TB patients showed separation of groups (Fig. 5B and C). However, when the same analysis was performed for TB against respiratory symptomatics, there was greater overlap between the groups (Fig. 5D and E), suggesting that this multiparameter approach using these variables alone will not provide a robust separation of patients with TB from respiratory symptomatics.

### Plasma MMP-8 differentiates patients with active pulmonary TB from symptomatic controls

**Fig 6:**
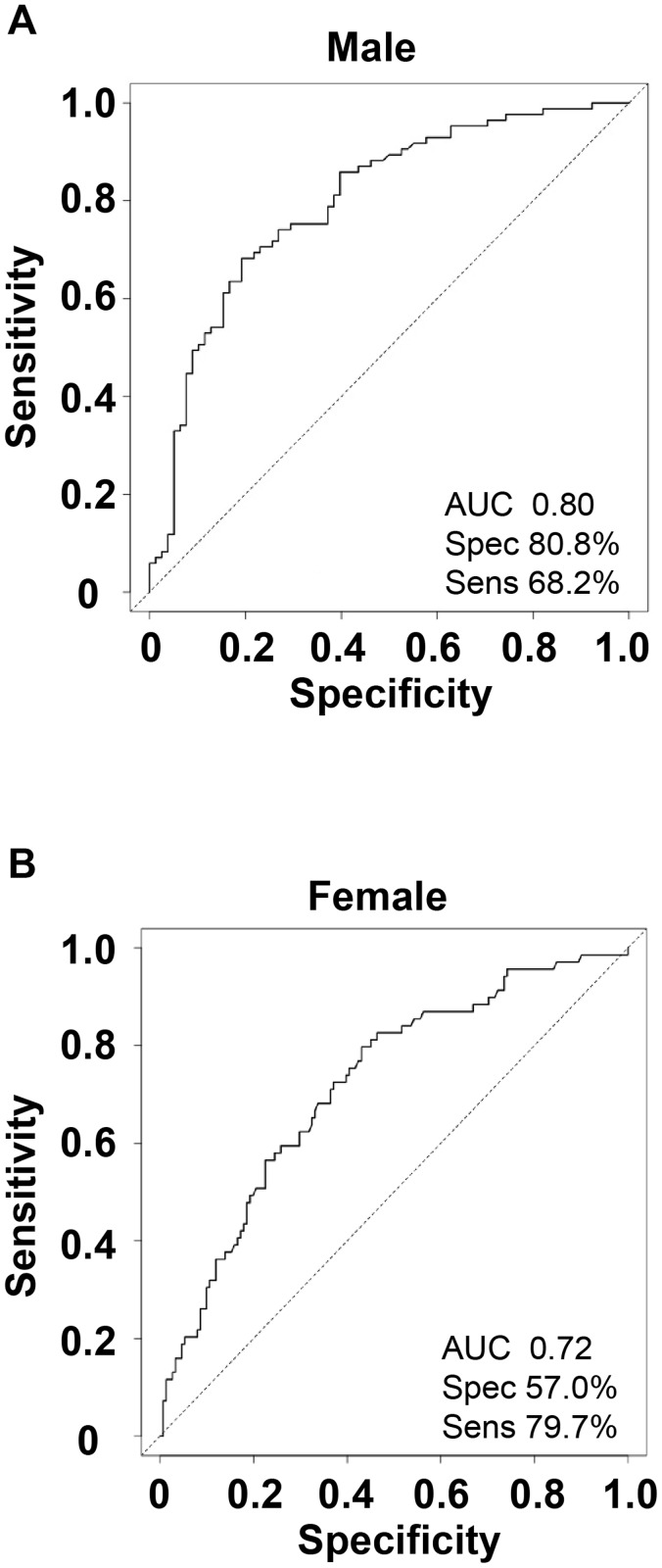
Plasma MMP-8 may discriminate patients with active pulmonary tuberculosis (TB) from symptomatic controls, but has better predictive value for men than women. A receiver operating characteristic (ROC) curve was plotted to investigate whether plasma MMP-8 concentrations discriminated between active pulmonary TB and respiratory symptomatics. The area under the curve (AUC) indicates predictive ability and is greater for men than women. AUC: area under curve, Spec: specificity, Sens: sensitivity.

We then performed receiver operating characteristic (ROC) analysis utilising MMP-8 as a single variable, since this had differed most significantly between patients with TB and respiratory symptomatics. The area under the curve (AUC) was 0.77 for all genders with a Youden’s criteria for optimal cut-off of MMP-8 = 6600 pg/ml with a specificity of 77.3% and sensitivity of 64.3% (Fig. B in S1 File). When ROC curves were plotted separately for males and females, plasma MMP-8 has a greater predictive ability for active TB in men compared to women (Fig. 6). For men the AUC is 0.80 with a Youden’s criteria of 6967 pg/ml and a higher specificity (80.8%) and sensitivity (68.2%.). For women the AUC is 0.72 with a lower Youden’s criteria of 3780 pg/ml and a lower specificity (57%) though a higher sensitivity (79.7%) than the ROC curve for the sexes combined or men alone.

## Discussion

We prospectively recruited and carefully characterised a cohort of 380 patients with active pulmonary TB, respiratory symptomatics (symptoms suspicious of TB but active disease excluded) and healthy controls. We report the first comprehensive analysis of circulating MMPs including the key comparator group of respiratory symptomatics. Two collagenases, MMP-1 and MMP-8, were elevated in active TB, but MMP-8 is specifically upregulated in TB compared to both symptomatics and healthy controls. Critically, MMP-8 was higher in males than females, potentially introducing an important confounder. Analysis of the sexes separately confirmed that MMP-8 is increased in active TB compared to symptomatics and controls. These data demonstrate that gender needs to be considered in investigation of TB immunopathology and novel diagnostic markers.

Previous studies have primarily assessed MMPs at the primary site of TB disease rather than in the circulation. MMPs-1, -2, -3 and-8 were increased in the induced sputum of patients with pulmonary TB and MMP-1 correlated with extent of radiological infiltration [15,16]. In contrast, in our study of circulating MMPs, the collagenase MMP-8 was most elevated in patients with TB compared to respiratory symptomatics, demonstrating that different immunological mediators predominate in each compartment. This suggests that measuring biomarkers at the site of disease and within the circulation may not identify the same pathological mediators, and that plasma analysis may not reflect the processes within the lung interstitium. MMP-8 is principally neutrophil-derived and its principal substrate is thought to be fibrillar collagen [27]. Collagen is the primary structural fibril of the pulmonary ECM and our findings support the hypothesis that excessive collagenolytic activity is critical to the tissue destruction that occurs in pulmonary TB [14]. Doxycycline, a widely available antibiotic, also inhibits MMP activity at sub-antimicrobial concentrations, and so our data further support the potential use of doxycycline as a host-directed therapy to limit immunopathology in TB [16]. The elevation of MMP-8, and the close association with MMP-9 on principal component analysis, suggests that neutrophils are the primary source of MMP-8, which are emerging as key mediators of pathology at time of TB diagnosis [20,21].

Plasma MMPs in tuberculosis have previously been investigated in smaller cohorts of patients. These studies have shown elevated MMP-8 and MMP-9, but none of these studies included respiratory symptomatic controls [28–30]. Also consistent with our findings, a recent study using aptamers for proteomic profiling of paired serum of TB patients at enrolment and after 8 weeks of TB chemotherapy showed that MMP-1, -8 and-9 fell during treatment [25]. The elevation of MMP-8 in TB compared to respiratory symptomatics contrasts with CRP, which has been reported to be more elevated in other respiratory conditions [31]. Investigation of plasma MMPs in other respiratory infections have shown that plasma MMP-9 is elevated in community acquired pneumonia compared to healthy controls and in two studies, MMP-9 was found to return to normal after antibiotic treatment [32–34]. In a study of hospital acquired pneumonia, MMP-8 and MMP-9 were elevated in both the bronchoalveolar lavage fluid and plasma of patients compared to healthy controls [35]. These findings are consistent with our data and suggest that MMPs may be peripheral markers of immune-mediated tissue damage.

We analyzed a cross-sectional study of patients with smear positive pulmonary disease and demonstrated differences in plasma MMPs compared to healthy controls. In addition, plasma MMP-7 was elevated in patients with latent TB relative to IGRA negative controls. MMP-7 has previously been implicated in TB-IRIS [36]. Large-scale population studies would be required to determine whether increased MMP-7 associates with an increased risk of developing active TB and is a marker of low-level disease activity. Important follow-up studies will be to compare pulmonary disease, where bacillary loads are high, with paucibacillary TB disease to determine whether MMPs diverge between these clinical presentations. In addition, a key subsequent study will be to determine the changes in plasma MMPs during TB treatment. We have previously shown that MMP concentrations in induced sputum correlate with treatment outcome [37], but are not aware of longitudinal studies of plasma MMPs during treatment.

Differences in plasma MMPs between men and women have been previously identified [23] and we confirmed in our study that MMP-8 was significantly higher in men with TB and this was not due to the potential confounder of delayed presentation. Men mount a greater and often more damaging inflammatory response to infection compared to women of reproductive age [38]. A proposed mechanism is that female sex hormones are protective [39] and female neutrophils have been found to express decreased MMP-9 during the period of the menstrual cycle when oestrogen levels are higher [40]. Clinical features of TB infection differ between men and women, although the biology is very poorly understood [7,11]. For example, men have a higher reported incidence of TB [9,10] and present more commonly with smear positive pulmonary disease [41]. Men have greater infiltrates on chest radiograph and more frequent haemoptysis [10]. In contrast, women more frequently develop lower zone pulmonary infiltrates [42]. These epidemiological observations demonstrate divergent immunopathology between sexes in human TB and are consistent with the hypothesis that differences in MMP expression causes greater tissue destruction in men [10]. The gender differences in plasma MMP-8 concentrations that we identify demonstrate a previously overlooked potential confounder in the assessment of novel TB diagnostic tests, which currently do not discriminate between men and women [24].

In summary, plasma MMP-1 and MMP-8 are elevated in active pulmonary TB, implicating excessive collagenase activity in TB immunopathology. MMP-8 is TB-specific and is higher in TB than in respiratory symptomatics. Plasma MMP-8 concentrations differ between men and women, demonstrating that gender must be considered in the investigation of TB pathology and development of novel TB diagnostics.

## Supporting Information

S1 File Table A. Plasma MMP concentrations are higher in men than women. Median levels and interquartile ranges (in brackets) for plasma matrix metalloproteinases (MMPs) analysed separately according to gender, for the entire cohort and separately by patient groups. Abbreviations: N/A—Not analysed. **Fig. A. Plasma MMP-7 concentrations are higher in healthy controls with a positive IGRA than those with a negative IGRA**. Plasma MMP concentrations analyzed by luminex array were compared by unpaired t-test. MMP-7 concentrations were significantly higher in individuals with a positive IGRA than a negative IGRA. **Fig. B. Plasma MMP-8 may discriminate patients with active pulmonary tuberculosis (TB) from symptomatic controls**. A receiver operating characteristic (ROC) curve was plotted to investigate whether plasma MMP-8 concentrations discriminated between active pulmonary TB and respiratory symptomatics. The area under the curve (AUC) was 0.77 indicating moderately high predictive ability. The optimal cut off was MMP-8 = 6600 pg/ml with specificity 77.3% and sensitivity 64.3%.
(PDF)

## References

[1] WHO (2014) Global tuberculosis report 2014.

[2] Udwadia ZF (2012) MDR, XDR, TDR tuberculosis: ominous progression. Thorax 67: 286–288. 10.1136/thoraxjnl-2012-201663 22427352

[3] Frieden TR, Sterling TR, Munsiff SS, Watt CJ, Dye C (2003) Tuberculosis. Lancet 362: 887–899. 13678977 10.1016/S0140-6736(03)14333-4

[4] Yoder MA, Lamichhane G, Bishai WR (2004) Cavitary pulmonary tuberculosis: The Holey Grail of disease transmission. Current Sci 86: 74–81.

[5] Kline SE, Hedemark LL, Davies SF (1995) Outbreak of tuberculosis among regular patrons of a neighborhood bar. N Engl J Med 333: 222–227. 7791838 10.1056/NEJM199507273330404

[6] Rodrigo T, Cayla JA, Garcia de Olalla P, Galdos-Tanguis H, Jansa JM, et al (1997) Characteristics of tuberculosis patients who generate secondary cases. Int J Tuberc Lung Dis 1: 352–357. 9432392

[7] Neyrolles O, Quintana-Murci L (2009) Sexual inequality in tuberculosis. PLoS Med 6: e1000199 10.1371/journal.pmed.1000199 20027210 PMC2788129

[8] Chen M, Kwaku AB, Chen Y, Huang X, Tan H, et al (2014) Gender and regional disparities of tuberculosis in Hunan, China. Int J Equity Health 13: 32 10.1186/1475-9276-13-32 24767610 PMC4013307

[9] Weiss MG (2006) Gender and tuberculosis: Cross-site analysis and implications of a multi-country study in Bangladesh, India, Malawi, and Colombia.

[10] Jimenez-Corona ME, Garcia-Garcia L, DeRiemer K, Ferreyra-Reyes L, Bobadilla-del-Valle M, et al (2006) Gender differentials of pulmonary tuberculosis transmission and reactivation in an endemic area. Thorax 61: 348–353. 16449260 10.1136/thx.2005.049452 PMC2104608

[11] Nhamoyebonde S, Leslie A (2014) Biological differences between the sexes and susceptibility to tuberculosis. J Infect Dis 209 Suppl 3: S100–106. 24966189 10.1093/infdis/jiu147

[12] Davidson JM (1990) Biochemistry and turnover of lung interstitium. Eur Respir J 3: 1048–1063. 2289553

[13] Vincenti MP, Brinckerhoff CE (2007) Signal transduction and cell-type specific regulation of matrix metalloproteinase gene expression: can MMPs be good for you? J Cell Physiol 213: 355–364. 17654499 10.1002/jcp.21208

[14] Elkington PT, D’Armiento JM, Friedland JS (2011) Tuberculosis immunopathology: the neglected role of extracellular matrix destruction. Sci Transl Med 3: 71ps76. 10.1126/scitranslmed.3001847 PMC3717269 21346167

[15] Elkington P, Shiomi T, Breen R, Nuttall RK, Ugarte-Gil CA, et al (2011) MMP-1 drives immunopathology in human tuberculosis and transgenic mice. J Clin Invest 121: 1827–1833. 10.1172/JCI45666 21519144 PMC3083790

[16] Walker NF, Clark SO, Oni T, Andreu N, Tezera L, et al (2012) Doxycycline and HIV infection suppress tuberculosis-induced matrix metalloproteinases. Am J Respir Crit Care Med 185: 989–997. 10.1164/rccm.201110-1769OC 22345579 PMC3359940

[17] Volkman HE, Pozos TC, Zheng J, Davis JM, Rawls JF, et al (2010) Tuberculous granuloma induction via interaction of a bacterial secreted protein with host epithelium. Science 327: 466–469. 10.1126/science.1179663 20007864 PMC3125975

[18] Hibbs MS, Bainton DF (1989) Human neutrophil gelatinase is a component of specific granules. J Clin Invest 84: 1395–1402. 2553773 10.1172/JCI114312 PMC304001

[19] Sepper R, Konttinen YT, Ding Y, Takagi M, Sorsa T (1995) Human neutrophil collagenase (MMP-8), identified in bronchiectasis BAL fluid, correlates with severity of disease. Chest 107: 1641–1647. 7781360 10.1378/chest.107.6.1641

[20] Berry MP, Graham CM, McNab FW, Xu Z, Bloch SA, et al (2010) An interferon-inducible neutrophil-driven blood transcriptional signature in human tuberculosis. Nature 466: 973–977. 10.1038/nature09247 20725040 PMC3492754

[21] Eum SY, Kong JH, Hong MS, Lee YJ, Kim JH, et al (2010) Neutrophils are the predominant infected phagocytic cells in the airways of patients with active pulmonary TB. Chest 137: 122–128. 10.1378/chest.09-0903 19749004 PMC2803122

[22] Mannello F (2008) Serum or plasma samples? The “Cinderella” role of blood collection procedures: preanalytical methodological issues influence the release and activity of circulating matrix metalloproteinases and their tissue inhibitors, hampering diagnostic trueness and leading to misinterpretation. Arterioscler Thromb Vasc Biol 28: 611–614. 10.1161/ATVBAHA.107.159608 18354094

[23] Mattey DL, Nixon NB, Dawes PT (2012) Association of circulating levels of MMP-8 with mortality from respiratory disease in patients with rheumatoid arthritis. Arthritis Res Ther 14: R204 10.1186/ar4042 23031278 PMC3580516

[24] Walzl G, Ronacher K, Hanekom W, Scriba TJ, Zumla A (2011) Immunological biomarkers of tuberculosis. Nat Rev Immunol 11: 343–354. 10.1038/nri2960 21475309

[25] De Groote MA, Nahid P, Jarlsberg L, Johnson JL, Weiner M, et al (2013) Elucidating novel serum biomarkers associated with pulmonary tuberculosis treatment. PLoS ONE 8: e61002 10.1371/journal.pone.0061002 23637781 PMC3630118

[26] Sandhu G, Battaglia F, Ely BK, Athanasakis D, Montoya R, et al (2012) Discriminating active from latent tuberculosis in patients presenting to community clinics. PLoS ONE 7: e38080 10.1371/journal.pone.0038080 22666453 PMC3364185

[27] Parks WC, Shapiro SD (2001) Matrix metalloproteinases in lung biology. Respir Res 2: 10–19. 11686860 10.1186/rr33 PMC59564

[28] Hrabec E, Strek M, Zieba M, Kwiatkowska S, Hrabec Z (2002) Circulation level of matrix metalloproteinase-9 is correlated with disease severity in tuberculosis patients. Int J Tuberc Lung Dis 6: 713–719. 12150484

[29] Sundararajan S, Babu S, Das SD (2012) Comparison of localized versus systemic levels of Matrix metalloproteinases (MMPs), its tissue inhibitors (TIMPs) and cytokines in tuberculous and non-tuberculous pleuritis patients. Hum Immunol 73: 985–991. 10.1016/j.humimm.2012.07.042 22820625 PMC3511911

[30] Seddon J, Kasprowicz V, Walker NF, Yuen HM, Sunpath H, et al (2013) Procollagen III N-terminal propeptide and desmosine are released by matrix destruction in pulmonary tuberculosis. J Infect Dis 208: 1571–1579. 10.1093/infdis/jit343 23922364 PMC3805234

[31] Choi CM, Kang CI, Jeung WK, Kim DH, Lee CH, et al (2007) Role of the C-reactive protein for the diagnosis of TB among military personnel in South Korea. Int J Tuberc Lung Dis 11: 233–236. 17263297

[32] Chiang TY, Shyu LY, Tsao TC, Chien MH, Tsao SM, et al (2012) Elevated plasma matrix metalloproteinase-9 protein and its gene polymorphism in patients with community-acquired pneumonia. Clin Chem Lab Med 50: 449–454. 10.1515/CCLM.2011.805 22107133

[33] Yang SF, Chu SC, Chiang IC, Kuo WF, Chiou HL, et al (2005) Excessive matrix metalloproteinase-9 in the plasma of community-acquired pneumonia. Clin Chim Acta 352: 209–215. 15653116 10.1016/j.cccn.2004.09.025

[34] Puljiz I, Markotic A, Cvetko Krajinovic L, Guzvinec M, Polasek O, et al (2012) Mycoplasma pneumoniae in adult community-acquired pneumonia increases matrix metalloproteinase-9 serum level and induces its gene expression in peripheral blood mononuclear cells. Med Sci Monit 18: CR500–505. 22847199 10.12659/MSM.883270 PMC3560704

[35] Hartog CM, Wermelt JA, Sommerfeld CO, Eichler W, Dalhoff K, et al (2003) Pulmonary matrix metalloproteinase excess in hospital-acquired pneumonia. Am J Respir Crit Care Med 167: 593–598. 12588713 10.1164/rccm.200203-258OC

[36] Tadokera R, Meintjes GA, Wilkinson KA, Skolimowska KH, Walker N, et al (2014) Matrix metalloproteinases and tissue damage in HIV-tuberculosis immune reconstitution inflammatory syndrome. Eur J Immunol 44: 127–136. 10.1002/eji.201343593 24136296 PMC3992843

[37] Ugarte-Gil CA, Elkington P, Gilman RH, Coronel J, Tezera LB, et al (2013) Induced Sputum MMP-1, -3 & -8 Concentrations during Treatment of Tuberculosis. PLoS ONE 8: e61333 10.1371/journal.pone.0061333 23613834 PMC3632571

[38] Guerra-Silveira F, Abad-Franch F (2013) Sex bias in infectious disease epidemiology: patterns and processes. PLoS ONE 8: e62390 10.1371/journal.pone.0062390 23638062 PMC3634762

[39] Marriott I, Huet-Hudson YM (2006) Sexual dimorphism in innate immune responses to infectious organisms. Immunol Res 34: 177–192. 16891670 10.1385/IR:34:3:177

[40] Smith JM, Shen Z, Wira CR, Fanger MW, Shen L (2007) Effects of menstrual cycle status and gender on human neutrophil phenotype. Am J Reprod Immunol 58: 111–119. 17631004 10.1111/j.1600-0897.2007.00494.x

[41] Diwan VK, Thorson A (1999) Sex, gender, and tuberculosis. Lancet 353: 1000–1001. 10459926 10.1016/S0140-6736(99)01318-5

[42] Aktogu S, Yorgancioglu A, Cirak K, Kose T, Dereli SM (1996) Clinical spectrum of pulmonary and pleural tuberculosis: a report of 5,480 cases. Eur Respir J 9: 2031–2035. 8902463 10.1183/09031936.96.09102031

