# Supplementary material for: Gender-Dependent Differences in Plasma Matrix Metalloproteinase-8 Elevated in Pulmonary Tuberculosis
Source: PLoS One. 2015 Jan 30;10(1):e0117605. doi: 10.1371/journal.pone.0117605 (PMC4312016; doi:10.1371/journal.pone.0117605)
Supplement: S1 File — Median levels and interquartile ranges (in brackets) for plasma matrix metalloproteinases (MMPs) analysed separately according to gender, for the entire cohort and separately by patient groups. Abbreviations: N/A—Not analysed. Fig. A. Plasma MMP-7 concentrations are higher in healthy controls with a positive IGRA than those with a negative IGRA. Plasma MMP concentrations analyzed by luminex array were compared by unpaired t-test. MMP-7 concentrations were significantly higher in individuals with a positive IGRA than a negative IGRA. Fig. B. Plasma MMP-8 may discriminate patients with active pulmonary tuberculosis (TB) from symptomatic controls. A receiver operating characteristic (ROC) curve was plotted to investigate whether plasma MMP-8 concentrations discriminated between active pulmonary TB and respiratory symptomatics. The area under the curve (AUC) was 0.77 indicating moderately high predictive ability. The optimal cut off was MMP-8 = 6600 pg/ml with specificity 77.3% and sensitivity 64.3%. (PDF) [file pone.0117605.s001.pdf]

		All	Healthy	Symptomatic	TB
MMP-1	Male	204 (9- 540)	7 (4-84)	344 (81-635)	287 (83-598)
	Female	83 (9-317)	10 (6-94)	202 (36-467)	178 (29-594)
MMP-3	Male	10134 (7302-14111)	10603 (7063 - 14968)	10725 (7836 - 14327)	9640 (7181 - 13789)
	Female	4848 (3352 - 6774)	5177 (3344 - 7517)	4978 (3440 - 6774)	4702 (3273 - 6508)
MMP-7	Male	34 (20 – 116)	N/A	N/A	N/A
	Female	3 (19 – 108)			
MMP-8	Male	6277 (3201 - 12381)	3413 (2409 - 6368)	4069 (2363 - 6889)	10622 (5381 - 17359)
	Female	4094 (2214 - 7725)	3125 (2040 - 6427)	3374 (2021 - 5722)	7043 (3909 - 13404)
MMP-9	Male	75150 (50272- 111266)	64786 (48935 - 107322)	77247 (46492 - 109487)	80411 (53645 - 117600)
	Female	55631 (38356 - 92406)	52659 (34556 - 82052)	53285 (35007 - 88313)	63861 (42572 - 108639)
MMP-10	Male	543 (376 – 788)	N/A	N/A	N/A
	Female	526 (364 – 764)			

Table A. Plasma MMP concentrations are higher in men than women. Median levels and interquartile ranges (in brackets) for plasma matrix metalloproteinases (MMPs) analysed separately according to gender, for the entire cohort and separately by patient groups. Abbreviations: N/A – Not analysed.

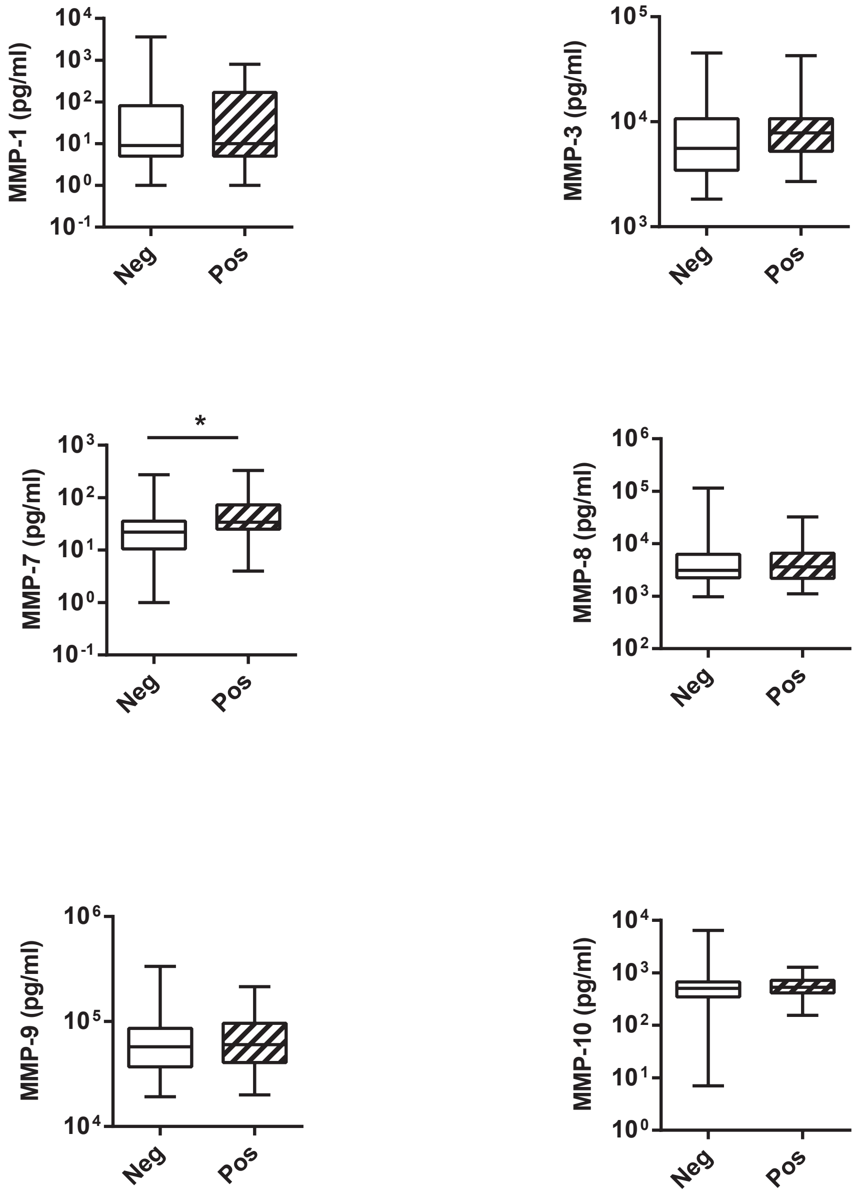

Figure A. Plasma MMP-7 concentrations are higher in healthy controls with a positive IGRA than those with a negative IGRA. Plasma MMP concentrations analyzed by luminex array were compared by unpaired t-test. MMP-7 concentrations were significantly higher in individuals with a positive IGRA than a negative IGRA.

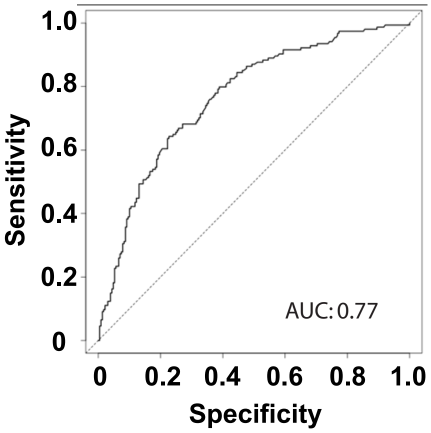

Figure B. Plasma MMP-8 may discriminate patients with active pulmonary tuberculosis (TB) from symptomatic controls. A receiver operating characteristic (ROC) curve was plotted to investigate whether plasma MMP-8 concentrations discriminated between active pulmonary TB and respiratory symptomatics. The area under the curve (AUC) was 0.77 indicating moderately high predictive ability. The optimal cut off was MMP-8 = 6600 pg/ml with specificity 77.3% and sensitivity 64.3%.
